
JOURNAL INFORMATION
==============================
NLM Title Abbreviation: ScientificWorldJournal
Journal ID: ScientificWorldJournal
NLM Journal ID: 101131163
Journal ID: TSWJ

The Scientific World Journal

ISSN: 2356-6140
EISSN: 1537-744X
Publisher: Wiley

ARTICLE INFORMATION
==============================
PMCID: PMC5901137
PMID: 29861697
DOI: 10.1100/tsw.2007.93
Article ID: 396054
Article version: 1
Subjects: Editorial

Glucocorticoid and Melanocortins: Special Issue

Getting Stephen J.
Department of Human and Health Sciences, School of Biosciences, University of Westminster, 115 New Cavendish Street London, W1W 6UW, UK
Publication date: 2007
Electronic publication date: 2007 Sep 1
Volume: 7
First page: 1463
Last page: 1464
Copyright: Copyright © 2007 Stephen J. Getting.
Copyright year: 2007
License: This is an open access article distributed under the Creative Commons Attribution License, which permits unrestricted use, distribution, and reproduction in any medium, provided the original work is properly cited.
License URL: https://creativecommons.org/licenses/by/3.0/

==============================
Editorial
Special Issue: Glucocorticoids and Melanocortins
*TheScientificWorldJOURNAL*, (2007) **7**, 1463–1464
ISSN 1537-744X; DOI 10.1100/tsw.2007.93

TheScientificWorldJOURNAL
www.thescientificworld.com

### Glucocorticoid and Melanocortins: Special Issue

Stephen J. Getting

*Senior Lecturer in Pharmacology, Department of Human and Health Sciences, School of Biosciences, University of Westminster, 115 New Cavendish Street, London, W1W 6UW, UK*

**E-mail:** s.getting@wmin.ac.uk

*Published September 1, 2007*

---

Glucocorticoids are amongst the most important and widely used drugs in the treatment of inflammatory diseases. The Nobel Prize for physiology or medicine was awarded to Kendall, Reichstein and Hench for their subsequent isolation and demonstration of biological efficacy of compound E (cortisone) or $ACTH_{1-39}$ (derived from POMC gene processing) in rheumatoid arthritis. Glucocorticoids have revolutionised how we treat inflammatory pathologies although the side effects associated with long term treatment can limit their use. Development of novel therapeutics based on POMC gene derived peptides (melanocortins), or unravelling the positive effects of glucocorticoids from detrimental ones, could be beneficial in the treatment of inflammatory diseases.

This issue explores the role that melanocortins and glucocorticoids play in modulating the host inflammatory response. Dr Stephen J. Getting and colleagues outline the role that the glucocorticoid inducible protein annexin 1 and POMC gene derived melanocortin peptides play in cardiovascular disease. Here they cite their ability to dampen down the release of pro-inflammatory mediators and subsequent migration of leukocytes into tissue, thus leading to a reduction in ischaemic damage within the heart. The anti-microbial efficacy of melanocortins and their ability to inhibit growth and replication of Staphylococcus aureas and Candida albicans is reviewed by Prof. Catania. Their research has opened up a new and exciting area for development of novel anti-microbial agents. The resolution of the host-inflammatory response is a novel and exciting area of research and has been reviewed by Dr. McColl and Prof. Rossi. In their review they have highlighted that glucocorticoids promote non-inflammatory phagocytosis of apoptotic cells. This is an important process in ensuring maintenance of cell membranes and limiting the damage to the host tissue. Unravelling the biological processes of apoptosis and development of drugs that favour this process, may be beneficial in chronic diseases that fail to resolve, which in turn lead to morbidity and death. Following on from this Dr. Scannell and Prof. Maderna have reviewed the unravelling of the biological effects of annexin 1 and lipoxins. The identification of a joint receptor ALXR and its ability to clear apopototic cells with a subsequent reduction in pro-inflammatory cytokines. This exciting line of research could lead to the development of pro-resolving therapeutics. A major problem in the treatment of patients with COPD and steroid insensitive asthmatics is management of their condition. Here, Dr. Ito reviews why patients fail to respond and the need to restore glucocorticoid efficacy. An understanding of how oxidative and nitrative stress exerts glucocorticoid resistance *via* reduction of corepressors or glucocorticoid receptor modification may allow for new therapeutics to be devised that will help in the treatment of these diseases. Finally, Dr. Catley has reviewed the role that dissociated glucocorticoids may play in modulating inflammation. The development of drugs which harness the trans-repression effects without the trans-activation side effects have been developed. However, these compounds work well *in vitro* whilst their biological efficacy is lost *in vivo*; therefore unravelling the molecular mechanisms involved could lead to drugs with an improved therapeutic index.

©2007 with author.

In conclusion harnessing the bodies own natural defences (melanocortins and annexin 1) or unravelling the positive attributes to glucocorticoids may lead to new therapies for the treatment of inflammatory pathologies. Finally, I would like to thank all the authors for their excellent contributions to this special issue and hope that the readers will find the reviews interesting and thought provoking.

**This article should be cited as follows:**

Getting, S.J. (2007). Glucocorticoid and melanocortins: special issue. *TheScientificWorldJOURNAL* **7**, 1463–1464. DOI 10.1100/tsw.2007.93.
